# Pollen production in olive cultivars and its interannual variability

**DOI:** 10.1093/aob/mcad163

**Published:** 2023-10-19

**Authors:** M Rojas-Gómez, J Moral, R López-Orozco, D Cabello, J Oteros, D Barranco, C Galán, C M Díez

**Affiliations:** Department of Agronomy, Agrifood Campus of International Excellence CeiA3, University of Cordoba, Rabanales Campus, Celestino Mutis Building, E-14071, Córdoba, Spain; Department of Botany, Ecology and Plant Physiology, Agrifood Campus of International Excellence CeiA3, University of Cordoba, Rabanales Campus, Celestino Mutis Building, E-14071, Córdoba, Spain; Department of Agronomy, Agrifood Campus of International Excellence CeiA3, University of Cordoba, Rabanales Campus, Celestino Mutis Building, E-14071, Córdoba, Spain; Department of Botany, Ecology and Plant Physiology, Agrifood Campus of International Excellence CeiA3, University of Cordoba, Rabanales Campus, Celestino Mutis Building, E-14071, Córdoba, Spain; Andalusian Inter-University Institute for Earth System IISTA, University of Cordoba, E-14071, Córdoba, Spain; Department of Agronomy, Agrifood Campus of International Excellence CeiA3, University of Cordoba, Rabanales Campus, Celestino Mutis Building, E-14071, Córdoba, Spain; Department of Botany, Ecology and Plant Physiology, Agrifood Campus of International Excellence CeiA3, University of Cordoba, Rabanales Campus, Celestino Mutis Building, E-14071, Córdoba, Spain; Andalusian Inter-University Institute for Earth System IISTA, University of Cordoba, E-14071, Córdoba, Spain; Department of Agronomy, Agrifood Campus of International Excellence CeiA3, University of Cordoba, Rabanales Campus, Celestino Mutis Building, E-14071, Córdoba, Spain; Department of Botany, Ecology and Plant Physiology, Agrifood Campus of International Excellence CeiA3, University of Cordoba, Rabanales Campus, Celestino Mutis Building, E-14071, Córdoba, Spain; Andalusian Inter-University Institute for Earth System IISTA, University of Cordoba, E-14071, Córdoba, Spain; Department of Agronomy, Agrifood Campus of International Excellence CeiA3, University of Cordoba, Rabanales Campus, Celestino Mutis Building, E-14071, Córdoba, Spain

**Keywords:** *Olea europaea*, pollen per anther, wind pollination, fruit load, androsterility, clustering, variability, biennial bearing

## Abstract

**Background and Aims:**

Olive (*Olea europaea* subsp. *europaea* var. *europaea*) is the most extensively cultivated fruit crop worldwide. It is considered a wind-pollinated and strictly outcrossing crop. Thus, elevated pollen production is crucial to guarantee optimum fruit set and yield. Despite these facts, the variability of pollen production within the cultivated olive has been scarcely studied. This study aimed to characterize this feature by analysing a representative set of worldwide olive cultivars.

**Methods:**

We evaluated the average number of pollen grains per anther in 57 principal cultivars over three consecutive years. We applied a standard generalized linear model (GLM) approach to study the influence of cultivar, year and the previous year’s fruit load on the amount of pollen per anther. Additionally, the *K*-means method was used for cluster analysis to group cultivars based on their pollen production capacity.

**Key Results:**

Pollen production per anther was highly variable among olive cultivars. The cultivar significantly accounted for 51.3 % of the variance in pollen production and the year for 0.3 %. The interaction between the two factors explained 8.4 % of the variance, indicating that not all cultivars were equally stable in producing pollen across the years. The previous year’s fruit load and its interaction with the year were significant, but barely accounted for 1.5 % of the variance. Olive cultivars were classified into four clusters according to their capacity to produce pollen. Interestingly, the fourth cluster was composed of male-sterile cultivars, which presumably share this character by inheritance.

**Conclusions:**

Pollen production per anther varied extensively within the cultivated olive. This variation was mainly driven by the cultivar and its interaction with the year. The differential capacity of olive cultivars to produce pollen should be considered not only for designing new orchards but also gardens where this species is used as an ornamental.

## INTRODUCTION

Cultivated olive (*Olea europaea* subsp. *europaea* var. *europaea*) is a species of essential historical, social and economic importance in the Mediterranean Basin. Cultivated olive is present in more than 40 countries and occupies a total area of 12.5 million hectares, being the most extensively planted fruit crop. Spain has the largest olive cultivated area and production, with ~2.5 million hectares and 35 % of olive oil production worldwide (FAOSTAT, 2020).

Olive cultivation is still primarily based on growing traditional olive cultivars under extensive rainfed conditions. However, the continued increase in demand for productivity has caused the olive sector to adopt new management strategies: harvest mechanization, super high-density systems, irrigation, and the use of new and more productive cultivars (Díez *et al.*, 2015; Rallo *et al.*, 2018; Lo Bianco *et al.*, 2021). The olive tree is also expanding beyond the Mediterranean boundaries in places such as China and the Americas, where its adaptation is occasionally uncertain (Ruiz *et al.*, 2019; Sánchez-Estrada and Cuevas, 2019).

It is thought that the selection of cultivars with high flower production has caused the olive tree pollination process to change over time, from entomophilous to anemophilous pollination (Rallo and Cuevas, 2007). Olive is currently considered a wind-pollinated crop and a strict outcrosser (Lavee *et al.*, 2002; Díaz *et al.*, 2006; Selak *et al.*, 2011), with few reported cases of auto-pollination, always giving rise to low fruit set (Farinelli *et al.*, 2008; Serrano *et al.*, 2008). Wind pollination requires the production of a very large amount of pollen during the flowering period to achieve successful fertilization (Cruden, 2000; Rallo and Cuevas, 2007). In fact, there is a positive correlation between annual pollen production and the subsequent olive yield (Galán *et al.*, 2004; Ribeiro *et al.*, 2007; Orlandi *et al.*, 2017), with the potential for fruit production forecasting.

Olive is an andromonoecious species with hermaphrodite flowers and a minor percentage of male flowers (Cuevas and Polito, 2004; Famiani *et al.*, 2019; Maniriho, 2022). Olive pollen grains are trizonocolporate with a reticulate exine (Trigo *et al.*, 2008), and their ultrastructure was the subject of extensive research during the 1980s and 1990s (Fernández and Rodríguez-García, 1988, 1989; Pacini and Juniper, 1979*a*, *b*; Rodríguez-García and Fernández, 1990). This pollen is highly allergenic and causes severe health problems in areas where the olive tree is present (Bousquet *et al.*, 1984; D’Amato, 1998). Twelve allergenic proteins in olive pollen have been identified (from Ole e 1 to Ole e 12) (Rodríguez *et al.*, 2007; Villalba *et al.*, 2014), whose expression and allergenic effects differ depending on the cultivar and the environment (Alché *et al.*, 2007; Quiralte *et al.*, 2007). In aerobiological studies, airborne olive pollen is considered as a whole because possible differences between cultivars (size, shape, apertures, thickness of the exine, and maximum length of the lumens) are not distinguishable under an optical microscope (Koubouris *et al.*, 2012; Ribeiro *et al.*, 2012; Messora *et al.*, 2017; Khaleghi *et al.*, 2019). Therefore, classifying olive cultivars according to their capacity to produce pollen could help to interpret allergy data and generate information for treatment and prevention (Galan *et al.*, 2007, 2013). Likewise, this information could lead to the selection of the most appropriate olive cultivars to be used as ornamental trees in public gardens due to their low or null pollen production.

An elevated pollen production capacity of olive cultivars is crucial to guarantee an optimum fruit set, especially in new olive-growing areas with no airborne olive pollen from surrounding orchards (Reale *et al.*, 2006; El-Soda *et al.*, 2017). In fact, even in traditional olive-growing areas, the use of several cultivars as pollen donors has been related to a fruit set increase (El-Soda *et al.*, 2017; Rallo *et al.*, 2018; Gencer et al., 2023). Despite these facts, there have been few studies characterizing the pollen production capacity of olive cultivars and its possible variability over years (Tous et al., 2004; Mazzeo *et al.*, 2014; Rojo *et al.*, 2015). Some studies showed high pollen production variability among olive cultivars but screened a restricted number of genotypes during a limited time (Tormo-Molina *et al.*, 1996; Cuevas and Polito, 2004; Ferrara *et al.*, 2007; Mazzeo *et al.*, 2014). Remarkably, the presence of several androsterile cultivars was also detected, but the pervasiveness, stability and cause of this character were not studied (Besnard *et al.*, 2000; Tous *et al.*, 2004).

Several factors, such as genotype and climatic and meteorological conditions, determine olive pollen production (Tous *et al.*, 2004; Rojo *et al.*, 2015). Specifically, temperature, water availability and solar radiation are some environmental variables causing alterations in olive vegetative and floral development (Rapoport, 2014; Benlloch-González *et al.*, 2018; Navas-Lopez *et al.*, 2019). In addition, flowering intensity and consequently pollen production per tree are strongly and negatively affected by the previous year’s fruit load, especially in species with a marked alternate bearing behaviour, such as olive (Famiani *et al.*, 2019). However, the possible effect of biennial bearing on the amount of pollen grains per anther has been scarcely tested in olive and other fruit species (Mazzeo *et al.*, 2014; Delgado *et al.*, 2021). Also, the applicability of pollen per anther production in yield forecasting is controversial in other species with studies of this aspect, such as grapevine (Fernández-González *et al.*, 2011; González-Fernández *et al.*, 2020).

Pollen per anther is one of the most robust metrics to estimate the pollen production of a species (Severova *et al.*, 2022). Pollen production per anther has been studied in fruit species, such as the apple tree (Javid and Rather, 2019; Delgado *et al.*, 2021) and mango (Pérez *et al.*, 2019), and in numerous forest species (Hidalgo *et al.*, 1999; Gómez-Casero *et al.*, 2004; Fernández-González *et al.*, 2020; Katz *et al.*, 2020) and grasses (Severova *et al.*, 2022; Prieto-Baena *et al.*, 2003). In olive, some studies have estimated pollen production per anther using different methodologies but always in a limited panel of genotypes (Orlandi *et al.*, 2003; Tous *et al.*, 2004; Cuevas and Polito, 2004; Ferrara *et al.*, 2007; Palasciano *et al.*, 2008; Aguilera and Valenzuela, 2012; Mazzeo *et al.*, 2014; Rojo *et al.*, 2015).

This study sought to examine the pollen production per anther of 57 of the most representative and economically significant olive cultivars worldwide over three years to (1) characterize the agronomical aptitude of the cultivars as pollen donors; (2) estimate the interannual variability in anther pollen production per cultivar as well as the influence of the genotype, the fruit load and the year; and (3) classify the olive cultivars according to their pollen production capacity.

## MATERIALS AND METHODS

### Location and plant material

This study was carried out in the World Olive Germplasm Bank (WOGB) – University of Cordoba (UCO) collection, located on the Campus of Rabanales in the UCO (Cordoba, Spain, 37°55ʹ56.5″N, 4°43ʹ13.3″W and 173 m a.s.l.). The WOGB includes more than 450 cultivars, and each cultivar is represented by two replicated trees. Olives were planted in 2011 and grown under drip irrigation (2000 m^3^ per hectare and year) at a spacing of 7 × 6 m. The entire collection was identified molecularly and morphologically (Trujillo *et al.*, 2013). The sanitary status of the olive plants was tested before planting (Morello *et al.*, 2016).

Pollen production was evaluated in 57 olive cultivars, with two trees per cultivar, during three consecutive years, from 2019 to 2021. The olive cultivars were selected according to their economic importance, genetic diversity (nuclear and chloroplastic) and distribution in the Mediterranean Basin (Besnard *et al.*, 2013; Díez *et al.*, 2015), with particular emphasis on cultivars of Spanish origin (Table 1). We also selected only those cultivars with both trees with the same bearing status, ‘on’ or ‘off’. Seven additional varieties from different areas of the Mediterranean Basin (‘Cerasuola’, ‘Chemlal de Kabilye’, ‘Escarabajuelo de Posadas’, ‘Frantoio’, ‘Koroneiki’, ‘Racimal de Jaén’ and ‘Sikitita’) were evaluated in 2020 and 2021 with the aim of expanding the genetic diversity of the studied cultivars.

**Table 1.** List of the 57 olive cultivars under evaluation and their countries of origin, genetic cluster and chlorotypes according to previous studies.

Cultivar name	Country	Genetic cluster^†^ (Díez *et al.*, 2015)	Chlorotype^‡^ (Besnard *et al.* 2013)
‘Adkam’	SYR	Q3	NA^¢^
‘Alameño Blanco’	SP	Q1	E1.1
‘Arbequina’	SP	Q2	E1.1
‘Arbosana’	SP	Mosaic	E1.1
‘Belluti’	TUR	Q3	E1.1
‘Blanqueta’	SP	Q2	E1.3
‘Bolvino’	SP	Q2	E1.1
‘Borriolenca’	SP	Q2	E3.1
‘Buidiego’	SP	Q1	NA
‘Canetera’	SP	Q2	NA
‘Carrasqueño de Porcuna’	SP	Q1	NA
‘Cerasuola’	ITA	NA	E3.2
‘Chemlal de Kabilye’	DZA	Mosaic	E3.2
‘Arracada de Aldover’	SP	Q2	E3.1
‘Curivell’	SP	Mosaic	NA
‘Empeltre’	SP	Q2	E1.1
‘Escarabajuelo de Posadas’	SP	Q1	E1.1
‘Farga’	SP	Q2	E3.1
‘Fishomi’	IRA	Q3	NA
‘Frantoio’	ITA	Q2	E1.1
‘Fulla de Salce’	SP	Mosaic	E3.1
‘Genotype 92’^*^	SP	NA	NA
‘Grosal Vimbodí’	SP	Q2	NA
‘Hemblasi’	SYR	Q3	E1.1
‘Hojiblanca’	SP	Q1	E1.1
‘Imperial de Jaén’	SP	Q1	NA
‘Joanenca’	SP	Mosaic	NA
‘Kato Drys’	CYP	Q3	E1.1
‘Koroneiki’	GRC	Q2	E1.1
‘Llumeta’	SP	Q2	E3.1
‘Loaime’	SP	Q1	NA
‘Manzanilla de Sevilla’	SP	Q1	E1.1
‘Marfil’	SP	NA	NA
‘Mari’	IRA	Q3	NA
‘Mawi’	SYR	Q3	NA
‘Menya’	SP	Q2	NA
‘Morona’	SP	Q1	NA
‘Morrut’	SP	Mosaic	E1.1
‘Palomar’	SP	Mosaic	NA
‘Perafort’	SP	Mosaic	NA
‘Picual’	SP	Q1	E1.1
‘Picual Blanco de Estepa’	SP	Q1	NA
‘Racimal de Jaén’	SP	Q1	E1.1
‘Royal de Sabiñán’	SP	Q2	NA
‘Safrawi’	SYR	Q3	NA
‘Sevillenca’	SP	Mosaic	E1.1
‘Shami’	SYR	Q3	E1.1
‘Shengue’	IRA	Q3	NA
‘Sikitita’	SP	NA	NA
‘Toffahi’	EGY	Mosaic	E1.2
‘Vallesa’	SP	Mosaic	NA
‘Vera’	SP	Q2	NA
‘Verdal de Manresa’	SP	Mosaic	NA
‘Verdala’	SP	Q1	E1.1
‘Verdale’	FRA	Mosaic	E1.1
‘Verdiell’	SP	Q2	E1.1
‘Villalonga’	SP	Mosaic	E1.3

NA, not available.

^*^Genotype 92: cultivar from UCO breeding programme.

^†^Genetic clusters: Q1, Western Mediterranean Basin; Q2, Central Mediterranean Basin; Q3, Eastern Mediterranean Basin and Mosaics = admixture between clusters.

^‡^Maternal genetic lineage.

The area of the collection is typically Mediterranean with dry summers and mild winters. Daily meteorological data were obtained from the European Climate Assessment and Dataset (ECA&D) project, which provides gridded data at a resolution of 0.1° latitude and 0.1° longitude. The data are available on the ECA&D website at http://www.ecad.eu/. The average annual temperature from 2019 to 2021 was 18.1 °C, with average high and low temperatures of 25.2 and 11.1 °C, respectively. The average annual precipitation was 323, 407 and 364 mm in 2019, 2020 and 2021, respectively (Supplementary Data Table S1).

### Fruit load and pollen production per anther

Fruit load intensity was assessed every year by estimating the percentage of the canopy covered by fruits using a visual scale from 0 to 3, where 0 indicated the absence of fruits; 1 indicated fruits covering ≤33 % of the canopy; 2 indicated fruits occupying 33–66 % of the canopy; and 3 indicated fruits covering >66 % of the canopy (Díez *et al.*, 2016).

The number of pollen grains per anther was estimated according to Cruden (1977). Briefly, for each tree we selected three different inflorescences immediately before anthesis and collected three mature hermaphrodite flowers per inflorescence in the same phenological stage; hence, we sampled 9 flowers per tree and 18 flowers per cultivar (two trees per cultivar were analysed). One undehisced anther from each mature flower was extracted and crushed in 100 µL of an aqueous fuchsin solution. Of this, 10 µL was deposited on a slide, and, once water had been evaporated with a heater, it was stained with fuchsin-stained glycerine gelatine to facilitate pollen grain counting according to Galan *et al.* (2007). Finally, the slides were sealed with coverslips and transparent nail polish. Pollen grain counts were performed under an optical microscope (Eclipse 80i, Nikon, Tokyo, Japan). Pollen grains were counted on each slide by scanning until reaching a minimum of 300 pollen grains. The total number of pollen grains per anther was calculated as the product of pollen grain counts (*P*_c_) and the total surface area of the coverslip (*S*_c_) divided by eyepiece diameter (*E*_d_), length of a scan (*L*_s_) and number of scans (*N*_s_).


$$\begin{array}{l} Total\;P_{c}\;\;\;per\;anther=\;\frac{S_{c}\;}{E_{d}\;\times L_{s}\times N_{s}}\times 10\; \end{array}$$

### Statistical analysis

The pollen count data per anther were analysed using Statistix software (version 10; Statistix, Tallahassee, FL, USA) and the R statistical software package. Given that our data did not satisfy the requirements of parametric tests (Shapiro‒Wilk test; *P* < 0.05), we used a standard generalized linear model (GLM) approach to study the influence of genotype and sampling year on the amount of pollen produced by different cultivars. The contribution of each factor (cultivar, year, previous-year fruit load and the interactions between factors) to the overall explained variance was calculated as the partial *ω*^2^ value (*ω*_p_^2^) (Yigit and Mendes, 2018). However, the partial *η*^2^ values (*η*_p_^2^) are also presented due to their wide use to estimate the proportion of population variance explained. Subsequently, we applied Bonferroni-corrected comparisons to examine the main effects and possible interactions between cultivars and the Kolmogorov–Smirnov test to determine the year’s impact on pollen production. Additionally, the *K*-means clustering method was used for cluster analysis to group cultivars based on their pollen production capacity. It should be noted that the two trees examined per cultivar were considered as a single individual since they were clones, and no statistically significant difference in pollen production was found between them.

## RESULTS

### Pollen production variability

Pollen production was highly variable among olive cultivars. Among the 57 evaluated cultivars, we found excellent pollen donors, while other cultivars did not produce pollen at all. For instance, ‘Alameño Blanco’ and ‘Mawi’, from Spain and Syria, respectively, were the largest pollen donors (>65 000 pollen grains per anther), along with the widely grown cultivar ‘Arbequina’. Other major cultivars, such as ‘Arbosana’ and ‘Manzanilla de Sevilla’ from Spain and ‘Frantoio’ from Italy, showed high pollen production (from 44 000 to 60 000 pollen grains per anther). In addition, important cultivars such as ‘Hojiblanca’ from Spain and ‘Koroneiki’ from Greece showed a medium pollen production capacity (44 402 and 43 150 pollen grains per anther, respectively). In contrast, some local cultivars, such as the Spanish ‘Royal de Sabiñán’ and ‘Racimal de Jaén’ cultivars, did not exceed the average value of 23 000 pollen grains per anther and had a coefficient of variation >90 % between years. Notably, 12 cultivars from Spain, one from Italy and another from Algeria behaved as androsterile and produced no pollen during the three years (Table 2). Therefore, we could consider the androsterility of these cultivars as a stable character.

**Table 2.** Summary of average pollen grain production per anther of the 57 olive cultivars screened during three consecutive crop seasons and their classification into significant groups (Bonferroni test; *P* < 0.05).

Cultivar	Average	Maximum	Minimum	Standard deviation	Standard error	CV (%)	Significant groups^*^
Androsterile cultivars^†^	0	0	0	0	0	0	A
‘Royal de Sabiñán’	15 946	69 240	0	18 861	2 567	118	AB
‘Racimal de Jaén’	22 752	85 320	0	21 054	3 509	93	ABC
‘Kato Drys’	33 632	99 120	0	23 443	3 190	70	ABCD
‘Shami’	36 786	78 360	3 860	16 279	2 215	44	ABCDE
‘Carrasqueño de Porcuna’	43 599	66 360	7 630	16 168	2 695	37	ABCDEF
‘Koroneiki’	43 150	81 000	0	22 679	3 780	53	ABCDEF
‘Sikitita’	43 121	80 280	90	20 811	3 468	48	ABCDEF
‘Joanenca’	40 323	136 680	0	33 254	4 525	82	BCDE
‘Adkam’	48 191	93 480	13 800	20 020	2 724	42	BCDEF
‘Belluti’	55 197	125 640	12 800	26 635	3 625	48	BCDEF
Bolvino’	50 136	100 920	9 990	21 471	2 922	43	BCDEF
‘Empeltre’	52 118	124 680	7 300	28 961	3 941	56	BCDEF
‘Escarabajuelo de Posadas’	58 634	131 400	12 210	34 388	8 105	59	BCDEF
‘Frantoio’	59 267	128 640	20 520	27 946	4 658	47	BCDEF
‘Hojiblanca’	44 402	197 400	11 970	29 125	3 963	66	BCDEF
‘Imperial de Jaén’	43 479	101 760	610	20 031	2 726	46	BCDEF
‘Loaime’	47 171	163 320	5 370	31 239	4 251	66	BCDEF
‘Picual Blanco de Estepa’	44 565	103 440	13 920	21 762	2 961	49	BCDEF
‘Mari’	47 490	109 560	13 230	20 636	2 808	43	BCDEF
‘Morona’	40 969	107 520	0	23 476	3 195	57	BCDEF
‘Picual’	44 818	82 680	11 430	20 968	2 853	47	BCDEF
‘Shengue’	44 387	102 000	5 970	19 770	2 690	45	BCDEF
‘Toffahi’	48 133	131 760	9 600	25 347	3 449	53	BCDEF
‘Verdala’	48 974	136 440	4 640	30 122	4 099	62	BCDEF
‘Verdale’	45 914	97 800	630	20 755	3 459	45	BCDEF
‘Verdiell’	48 129	141 840	9 630	24 918	3 391	52	BCDEF
‘Villalonga’	52 117	254 400	0	43 073	5 862	83	BCDEF
‘Arbosana’	59 012	226 080	4 600	49 973	6 800	85	CDEF
‘Blanqueta’	60 574	146 880	11 910	30 503	4 151	50	CDEF
‘Buidiego’	58 910	112 440	13 320	25 477	3 467	43	CDEF
‘Fishomi’	60 746	139 080	18 120	30 710	4 179	51	CDEF
‘Hemblasi’	57 249	116 640	11 490	25 777	3 508	45	CDEF
‘Manzanilla de Sevilla’	58 072	121 920	9 390	30 214	4 112	52	CDEF
‘Marfil’	61 167	185 760	14 760	29 499	4 014	48	CDEF
‘Menya’	62 121	141 000	7 370	30 222	4 113	49	CDEF
‘Morrut’	56 956	119 760	12 570	27 765	3 778	49	CDEF
‘Perafort’	57 957	116 760	2 610	25 417	3 459	44	CDEF
‘Safrawi’	61 018	183 840	13 200	34 268	4 663	56	CDEF
‘Sevillenca’	56 345	176 640	10 710	32 868	4 473	58	CDEF
‘Grossal Vimbodí’	67 912	184 800	6 660	35 636	4 849	52	DEF
‘Arbequina’	76 561	155 760	9 900	33 544	4 565	44	EF
‘Mawi’	76 744	221 760	18 060	39 358	5 356	51	EF
‘Alameño Blanco’	80 701	267 840	18 120	48 715	6 629	60	F

^*^The letters indicate the homogeneous groups according to Bonferroni correction (*P* < 0.05). Different letters indicate significant differences according to Bonferroni correction.

^†^Non-pollen-producing cultivars: ‘Borriolenca’, ‘Canetera’, ‘Cerausola’, ‘Chemlal de Kabylye’, ‘Arracada de Aldover’, ‘Curivell’, ‘Farga’, ‘Fulla de Salce’, ‘Genotype 92’, ‘Llumeta’, ‘Palomar’, ‘Vallesa’, ‘Vera’, ‘Verdal de Manresa’.

**Fig. 1.**
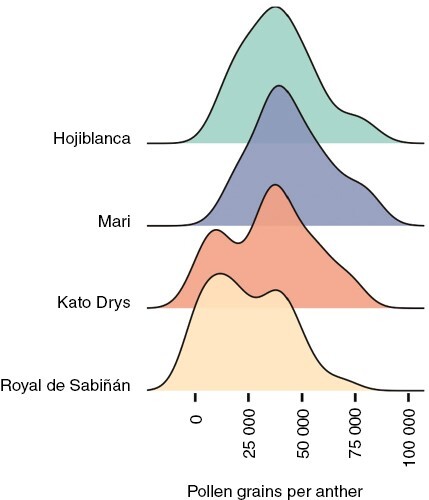
Distribution of olive pollen production during three consecutive years represented as density charts. Cultivars ‘Hojiblanca’ and ‘Mari’ presented a normal distribution, while ‘Kato Drys’ and ‘Royal de Sabiñán’ showed a bimodal distribution.

Generally, the amount of pollen per anther and cultivar followed a normal distribution (Fig. 1). However, that of ‘Joanenca’, ‘Kato Drys’, ‘Royal de Sabiñán’, ‘Racimal de Jaén’ and ‘Villalonga’ showed a bimodal distribution due to the production of contrasting amounts of pollen per anther, i.e. anthers with large amounts of pollen (>100 000 pollen grains) and others with low or null pollen (<1000 pollen grains). Other cultivars, such as ‘Koroneiki’ and ‘Morona’, occasionally showed some anthers with no pollen, but this phenomenon was not pervasive. Remarkably, ‘Verdale’ and ‘Carrasqueño de Porcuna’ did not produce flowers in 2019 and 2020, respectively, due to a marked alternate bearing behaviour.

### Factors contributing to pollen production variability

In our study, we evaluated and quantified the possible effect of cultivar (genotype), year, previous-year fruit load (fruit load) and the interaction between these factors on pollen production. To do so, we applied a GLM analysis considering cultivar, year, fruit load and the interactions between them as categorical variables (Table 3).

**Table 3.** Generalized linear model analysis of pollen production per anther showing the percentages of variance explained by the evaluated factors [cultivar, interannual variability (year), previous year’s load (fruit load) and their interactions] and their significance level.

Factor	*ω*_p_^2^ ( %)	*η*_p_^2^ ( %)	*F* ratio	*P*-value
Cultivar	51.3	53.2	53.91	<0.001
Year	0.3	0.1	9.53	<0.01
Fruit load	0.1	0	6.48	<0.05
Cultivar × year	8.4	8.5	5.56	<0.001
Cultivar × fruit load	1.3	3.1	1.8	<0.01
Year × fruit load	0	0	0.31	>0.05
Cultivar × year × fruit load	1.8	2.6	4.59	<0.001
Residual	36.8	32.5		

*ω*_p_^2^ and *η*_p_^2^ are the percentages of pollen production variability explained by each factor and their interaction.

*F* ratio is the variation between samples/variation within the samples.

Predictably, cultivar was the most determinant factor (GLM test; *P* < 0.001), explaining >51.3 % of the total variation (*ω*_p_^2^). The effects of year and fruit load on pollen production were also statistically significant (GLM test; *P* < 0.01 and *P* < 0.05, respectively) but accounted for <~0.5 % of the variation (*ω*_p_^2^). The cultivar × year interaction (GLM test; *P* < 0.001) accounted for ~8 % of the variation (*ω*_p_^2^), indicating that the cultivars were not homogeneously affected by year in terms of their capacity to produce pollen. For instance, ‘Carrasqueño de Porcuna’ showed stable pollen production across the three years, while others, such as ‘Arbosana’, produced contrasting amounts of pollen depending on the year. Similarly, the interactions cultivar × fruit load and the three-way interaction cultivar × year × fruit load were significant (GLM test; *P* < 0.01 and *P* < 0.001, respectively), but barely contributed ~3 % to the total variation (Supplementary Data Fig. 1).

**Fig. 2.**
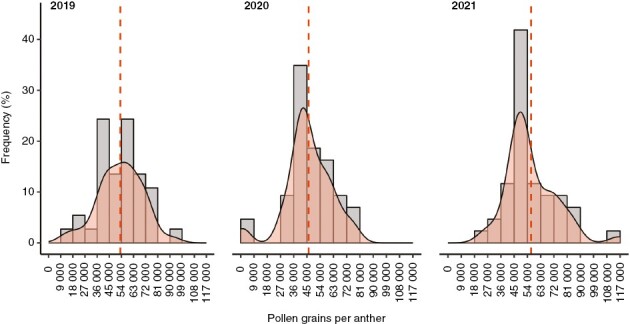
Frequency distribution of pollen production shown by olive cultivars during the three evaluated years. The relative frequency was calculated for the average of all the repetitions for the same cultivar. Each graph shows the mean number of pollen grains per anther (red dashed line; 2019 = 53 228 ± 16 339; 2020, 46 118 ± 15 757; 2021, 56 469 ± 17 052) and density (light grey area).

Global pollen production differed significantly between 2020 and 2021 (Kolmogorov–Smirnov test; *P* value < 0.01) but neither between 2019 and 2020, nor 2019 and 2021 (Kolmogorov–Smirnov test; *P* value > 0.05). The highest frequency of pollen production per anther was recorded in 2021, the lowest frequency was recorded in 2020, and the frequency in 2019 was intermediate, with average values of 42 040 ± 36 800, 34 770 ± 31 480 and 38 560 ± 36 910 pollen grains per anther, respectively. The significant effect of year on pollen production was evident when we plotted the frequency distribution of the average pollen production per anther for the whole dataset of cultivars (Fig. 2).

### Classification of cultivars according to their pollen production

**Fig. 3.**
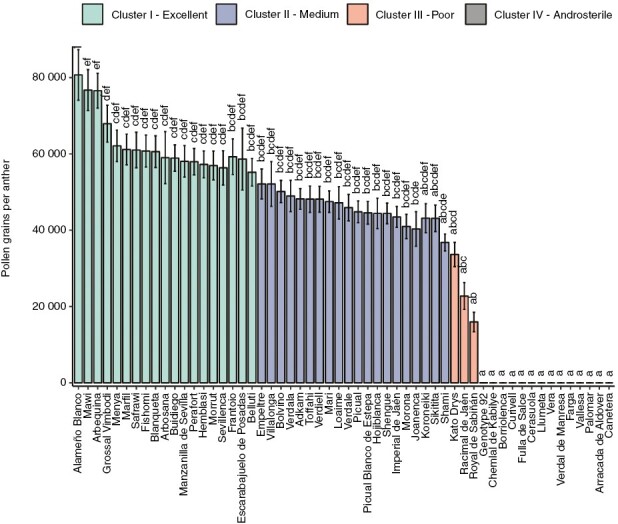
Frequency distribution of average pollen production of the cultivars during the three evaluated years (2019–21). Relative frequency was calculated for the average of all repetitions for the same cultivar. Different colours show the three homogeneous clusters according to their pollen production capacity: Cluster I in blue, Cluster II in green and Cluster III in red. Letters indicate significant differences after Bonferroni correction (*P* < 0.05).

Given that cultivar was the most crucial factor determining pollen production, we classified the cultivars according to this feature. To this end, we applied a hierarchical cluster analysis using mean values of pollen production. According to the results, we categorized the olive genotypes into four main clusters: excellent (Cluster I), medium (Cluster II), poor (Cluster III) and androsterile pollen donors (Cluster IV) (Table 4; Fig. 3).

**Table 4.** Olive cultivar classification according to pollen grain production per anther, name and group size, and summary statistics.

Cluster	Cultivar	*n*	Mean	s.d.	Min	Max
I	‘Alameño Blanco’, ‘Arbequina’, ‘Arbosana’, ‘Belluti’, ‘Blanqueta’, ‘Buidiego’, ‘Escarabajuelo de Posadas’, ‘Fishomi’, ‘Frantoio’, ‘Grossal Vimbodí’, ‘Hemblasi’, ‘Manzanilla de Sevilla’, ‘Marfil’, ‘Mawi’, ‘Menya’, ‘Morrut’, ‘Perafort’, ‘Safrawi’, ‘Sevillenca’	19	63 270	7490	55 190	80 700
II	‘Adkam’, ‘Bolvino’, ‘Carrasqueño de Porcuna’, ‘Empeltre’, ‘Hojiblanca’, ‘Imperial de Jaén’, ‘Joanenca’, ‘Koroneiki’, ‘Loaime’, ‘Mari’, ‘Morona’, ‘Picual’, ‘Picual Blanco de Estepa’, ‘Shami’, ‘Shengue’, ‘Sikitita’, ‘Toffahi’, ‘Verdala’, ‘Verdale’, ‘Verdiell’, ‘Villalonga’	21	45 610	3830	36 780	52 120
III	‘Kato Drys’, ‘Racimal de Jaén’, ‘Royal de Sabiñán’	3	24 110	8910	15 950	33 630
IV	‘Borriolenca’, ‘Canetera’, ‘Cerasuola’, ‘Chemlal de Kabylye’, ‘Arracada de Aldover’, ‘Curivell’, ‘Farga’, ‘Fulla de Salce’, ‘Genotype 92’, ‘Llumeta’, ‘Palomar’, ‘Vallesa’, ‘Vera’, ‘Verdal de Manresa’	14	0	0	0	0

**Fig. 4.**
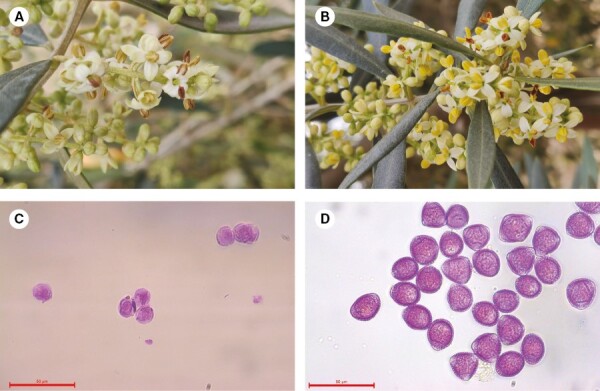
Inflorescences and undeveloped pollen grains stained with fuchsin (scale bar = 50 μm) in the androsterile cultivar ‘Vera’ (A and C) and in the excellent pollen donor ‘Arbequina’ (B and D).

Cluster I comprised 19 cultivars (~33 %) and showed the highest average count of pollen grains per anther (from 55 190 to 80 700); ‘Arbequina’ and ‘Arbosana’, from Spain, ‘Belluti’ from Turkey and ‘Fishomi’ from Iran were included in this cluster. Cluster II was the most abundant group, with 21 cultivars (~37 %), such as ‘Villalonga’ and ‘Empeltre’, which produced the most pollen (52 117 ± 43 073 and 52 118 ± 28 961 pollen grains per anther, respectively), and important cultivars, such as ‘Mari’ from Iran and ‘Toffahi’ from Egypt. Cluster III was composed of ‘Kato Drys’, ‘Racimal de Jaén’ and ‘Royal de Sabiñán’, which were considered poor pollen donors because they had the lowest pollen counts, with an average value of 24 110 ± 8910 pollen grains/anther. The 14 androsterile cultivars (‘Borriolenca’, ‘Canetera’, ‘Cerasuola’, ‘Chemlal de Kabylye’, ‘Arracada de Aldover’, ‘Curivell’, ‘Farga’, ‘Fulla de Salce’, ‘Genotype 92’, ‘Llumeta’, ‘Palomar’, ‘Vallesa’, ‘Vera’ and ‘Verdal de Manresa’) (~25 %) produced no pollen in any of the evaluated years and were grouped into Cluster IV. This group had normal flowers, but in many cases their anthers showed a brownish colour instead of being bright yellow (Fig. 4), a characteristic previously mentioned by Riera (1950).

We also tested the consistency of these groups over the years. To this end, the *K*-means algorithm was applied to generate groups considering the average values of pollen production per year. We found that ~37 % of the varieties remained in the same group among the three years, and ~44 % did so in two years. It is worth mentioning that these latter cultivars changed between contiguous categories; for instance, ‘Alameño Blanco’ was classified in Cluster II in 2019 and in Cluster I in 2020 and 2021. On the other hand, the classification of ~19 % of the cultivars was quite variable, with them being eventually included in the three different production categories over the study period. These cultivars strongly contributed to the significant effect of the cultivar × year interaction.

## DISCUSSION

### Pollen production variability

This study presents the most extensive and exhaustive characterization of pollen production of different olive cultivars. Our results showed that olive has a large degree of variability in its capacity to produce pollen, with genotype being the most determinant factor for this feature, but it is also affected by other factors, such as biennial bearing. This result is in agreement with those of previous studies, which revealed marked differences in pollen production between olive cultivars but considered a restricted number of cultivars and evaluated years (Rallo and Cuevas, 2007; Mazzeo *et al.*, 2014). We found cultivars producing on average >60 000 pollen grains per anther, such as ‘Blanqueta’, ‘Safrawi’ and ‘Grossal Vimbodí’, while others were unable to produce half this amount, such as ‘Royal de Sabiñán’, ‘Racimal de Jaén’ and ‘Kato Drys’. Notably, the pollen production of most cultivars was closer to the upper classification extreme and could be considered medium-high (54 440 ± 5660 pollen grains per anther).

Our average pollen counts per anther were lower than those previously reported when applying the same methodology (Tormo-Molina *et al.*, 1996), but the study used only three olive trees of uncertain status, cultivated or wild. On the other hand, our counts were in line with those of other previous studies (Orlandi *et al.*, 2003; Cuevas and Polito, 2004; Aguilera and Valenzuela, 2012; Rojo *et al.*, 2015), which applied a different methodology based on measuring the pollen of a set of anthers from different flowers as samples (Dafni, 1992). Other studies showed higher counts than ours, but these combined the latter methodology with the use of a Bürker haemocytometer for pollen counting instead of an optical microscope (Ferrara *et al.*, 2007; Palasciano *et al.*, 2008; Mazzeo *et al.*, 2014; Rojo *et al.*, 2015).

Three cultivars, ‘Kato Drys’, ‘Royal de Sabiñán’ and ‘Racimal de Jaén’, presented a significantly low average pollen count. Moreover, in these cultivars we observed a heterogeneous pollen distribution per anther, which gave rise to a bimodal distribution (Fig. 1). This feature was caused by some anthers having an average pollen count of ~24 000 pollen grains per anther, while others showed a reduced or null amount. This phenomenon is called partial male sterility, and it was previously reported as a genetic feature in other olive cultivars, such as ‘Tanche’ (Besnard *et al.*, 2000) and ‘Swan Hill’ (Fernandez-Escobar and Martin, 1986). This phenomenon was also observed in other plant species, such as sunflower, maize, and citrus (Putt and Heiser, 1966; Weider *et al.*, 2009; Raveh *et al.*, 2020).

The most extreme deviation regarding pollen production was observed in 14 cultivars with no pollen counts over the three years of evaluation. Twelve of them were from northeastern Spain and, presumably, shared genetic ancestry according to their phylogenetic relationships (Besnard *et al.*, 2013; Díez *et al.*, 2015). Given these facts, the absence of pollen production could be a character shared by descendants, although further genetic and physiological studies are necessary to corroborate this hypothesis. Most of these cultivars (except ‘Borriolenca’ and ‘Genotype 92’) were previously reported as androsterile elsewhere (Amane *et al.*, 1999; Besnard *et al.*, 2000; Tous *et al.*, 2004). Other cases of olive cultivars with male sterility were also cited in France for the ‘Lucques’ and ‘Tanche’ cultivars, in Algeria for ‘Aaroun’ and ‘Hamra’, and in Tunisia for ‘Zarazi’ (Besnard *et al.*, 2000; Cavallotti *et al.*, 2003). It is worth mentioning that the complete reference genome for the olive tree is from one of the androsterile cultivars, ‘Farga’ (Cruz *et al.*, 2016). Therefore, this feature should be seriously taken into account before considering this cultivar as the standard for the species.

### Factors contributing to pollen production variability

Cultivar (genotype) accounted for the largest part of the variability observed in pollen production between olive cultivars, contributing 51.3 % of the variance. A major influence of genotype on pollen production per anther was reported but not quantified in other species, such as haskap berries, sweet cherry and grapes (Bieniasz *et al.*, 2019; Dziedzic *et al.*, 2019; Kowalczyk *et al.*, 2022).

In this study, year had a significant but much weaker influence on the variance, contributing barely 0.3 %. Annual fluctuations in environmental conditions affect pollen production (Raja *et al.*, 2019; Yu *et al.*, 2019); indeed, changes in temperature and precipitation might cause irregularities in microsporogenesis and ultrastructural changes in pollen grains (Chaturvedi *et al.*, 2021; Lamin-Samu *et al.*, 2021; Ullah *et al.*, 2022). For instance, olive pollen develops during the 6–4 weeks prior to flowering (approximately the first week of May); therefore, the temperatures during this time (March and April) are determinants of pollen production (Pacini *et al.*, 1985; Fernández and Rodríguez-García, 1988; Fodale *et al.*, 1994; Ateyyeh *et al.*, 2000). Given the limited number of years evaluated in this study, we were unable to establish any robust relationships between pollen production and meteorological variables. The year with the highest average pollen production (2021) also had the highest accumulated precipitation from January to April, but we did not detect any other remarkable meteorological features able to explain the observed pollen variation between years.

Interestingly, environmental factors do not have the same impact on all cultivars (Lavee, 2007; Medina-Alonso *et al.*, 2020; Fernández-González *et al.*, 2021). In our study, the interaction cultivar × year accounted for 8.4 % of the total variance, considered a medium effect in multifactorial experiments (Cohen, 1988). In line with this result, not all the cultivars were equally stable in pollen production over the years; some of them, such as ‘Adkam’, showed a coefficient of variation (CV) of 42 %, while others, such as ‘Arbosana’ and ‘Villalonga’, showed double this number (CV ≈ 80 %). Longer time series are required to assess the specific cause of this unequal variation. We hypothesize that cultivars with larger CVs have a more unstable behaviour, and thus they could be more susceptible to climate change. However, there are other variables affecting the performance of the tree, such as its nutritional status, which were considered in this study. Remarkably, ‘Joanenca’ and ‘Villalonga’ were reported as androsterile by Tous *et al.* (2004); however, we classified them as partially male-sterile because they showed pollen production, although it was reduced and heterogeneous. This phenomenon, reported in other species, might be triggered by specific climatic conditions, such as heat stress (Begcy *et al.*, 2019; Li *et al.*, 2022).

Biennial bearing affects the number of fruits, although previously it was found to affect the number of flowers (Rallo and Cuevas, 2007; Seifi *et al.*, 2015; Maniriho, 2022) and of pollen grains per anther (Mazzeo *et al.*, 2014). We confirmed this latter observation; the previous year’s fruit load (GLM test; *P* < 0.05), as well as its interactions with cultivar and cultivar × year, were significant (GLM test; *P* < 0.01 and *P* < 0.001, respectively). However, the contribution of these categorical variables to the overall variance barely exceeded 3 %. Resource limitation before inflorescence formation caused by a previous heavy fruit load affects the number of inflorescences, the number of flowers per inflorescence, the floral quality and fruit set (Lavee et al., 1996; Cuevas and Polito, 2004; Rallo and Cuevas 2007). According to Mazzeo *et al.* (2014), olive compensates for the lower pollen production in ‘off’ years by increasing pollen viability. However, studies in other fruit species, such as apple (Delgado *et al.*, 2021) and apricot (Gallotta *et al.*, 2014), did not find significant differences in pollen production per anther between ‘on’ and ‘off’ years. These discrepancies highlight the need for further research to fully understand the effect of biennial bearing on pollen production in perennial fruit crops.

### Classification of cultivars according to their pollen production

The major and more significant effect of cultivar on pollen production allowed us to apply a *K*-means clustering analysis to classify the cultivars according to this feature. As a result, we classified the evaluated cultivars into four groups: excellent (Cluster I), medium (Cluster II), poor (Cluster III) and androsterile (Cluster IV) pollen donors. We did not detect any previously documented genetic relationship between the cultivars included in every cluster, other than the one mentioned above for androsterile cultivars.

Cluster I included 19 cultivars characterized by the highest pollen production, such as ‘Arbequina’ and ‘Arbosana’, in agreement with the findings of previous studies that already highlighted their outstanding capacity as pollen donors (Ferrara *et al.*, 2007). These cultivars are the most propagated by the nursery industry because of their excellent performance in super-intensive orchards, which constitute the majority of new plantations worldwide (Díez *et al.*, 2016; Rallo *et al.*, 2018). Cluster II included 21 cultivars with medium pollen production, comprising widely spread cultivars, such as ‘Picual’, with >1 000 000 ha in Spain, and ‘Koroneiki’, the main cultivar in Greece (Rallo *et al.*, 2018). Cluster III was composed of ‘Kato Drys’, the main crop in Cyprus, and the Spanish cultivars ‘Racimal de Jaén’ and ‘Royal de Sabiñán’, with little diffusion outside their presumed areas of origin.

Finally, Cluster IV included androsterile cultivars such as ‘Farga’ and ‘Chemlal de Kabylye’, cultivars found in Spain and Algeria, despite its inability to produce pollen. On the other hand, ‘Genotype 92’ was a chance seedling from the UCO breeding programme, being androsterile but also presenting staminate flowers and therefore being unable to produce fruits. These two characteristics make ‘Genotype 92’ a highly valuable variety in an ornamental context (Ashok and Velmurugan, 2020; Culley *et al.*, 2022).

Our classification of olive cultivars according to their aptitude as pollen donors is essential for designing new plantations, especially in non-traditional production countries. In this context, the compatibility between cultivars must also be considered to guarantee the plantation’s success. Olive shows pollen–pistil self-incompatibility, with cultivars apparently grouped into two proposed compatibility groups (i.e. cross-pollination between individuals of the same group causes a response of incompatibility) (Saumitou-Laprade *et al.*, 2010, 2017; Alagna *et al.*, 2019); however, this hypothesis is still controversial (Farinelli *et al.*, 2018; Breton *et al.*, 2021).

Our results contribute to increasing our knowledge about olive cultivar performance, giving rise to new questions regarding olive pollen development, fruit set and floral physiology. Indeed, further studies are needed to complete information regarding possible differences in pollen quality parameters (such as viability) between cultivars, as it was outlined by other authors (Rejón *et al.*, 2010; Ribeiro *et al.*, 2012). Additionally, we aimed to determine the physiological cause of the androsterility observed in some cultivars and to reveal the effect of elevated temperatures on pollen production, pollen quality and possible differences between cultivars.

### Conclusions

We found important and significant variability in pollen production per anther within cultivated olive. In fact, genotype accounted for the largest proportion of the variance, followed by year and the previous year’s fruit load. Remarkably, the interaction between the three factors was significant, particularly between cultivar and year, indicating that the stability of pollen production was not a pervasive character. Our results allowed us to classify the olive cultivars into four clusters according to their capacity to produce pollen. Interestingly, the fourth group was composed of androsterile cultivars, which could share a common origin. This study provides novel and useful information for the establishment of new olive plantations and creates new lines of inquiry for the characterization of olive pollen development, quality and floral physiology.

## SUPPLEMENTARY DATA

Supplementary data are available at *Annals of Botany* online and consist of the following. Table S1: pollen production and summary of the air temperatures and precipitation during the months prior to flowering in the three evaluated years. Figure S1: distribution of cultivar pollen production per anther according to their previous-year yield.

mcad163_suppl_Supplementary_Figure_S1

mcad163_suppl_Supplementary_Table_S1
