# Supplementary figures and images for: Pollen production in olive cultivars and its interannual variability
Source: Ann Bot. 2023 Oct 19;132(6):1145–58. doi: 10.1093/aob/mcad163 (PMC10809056; doi:10.1093/aob/mcad163)

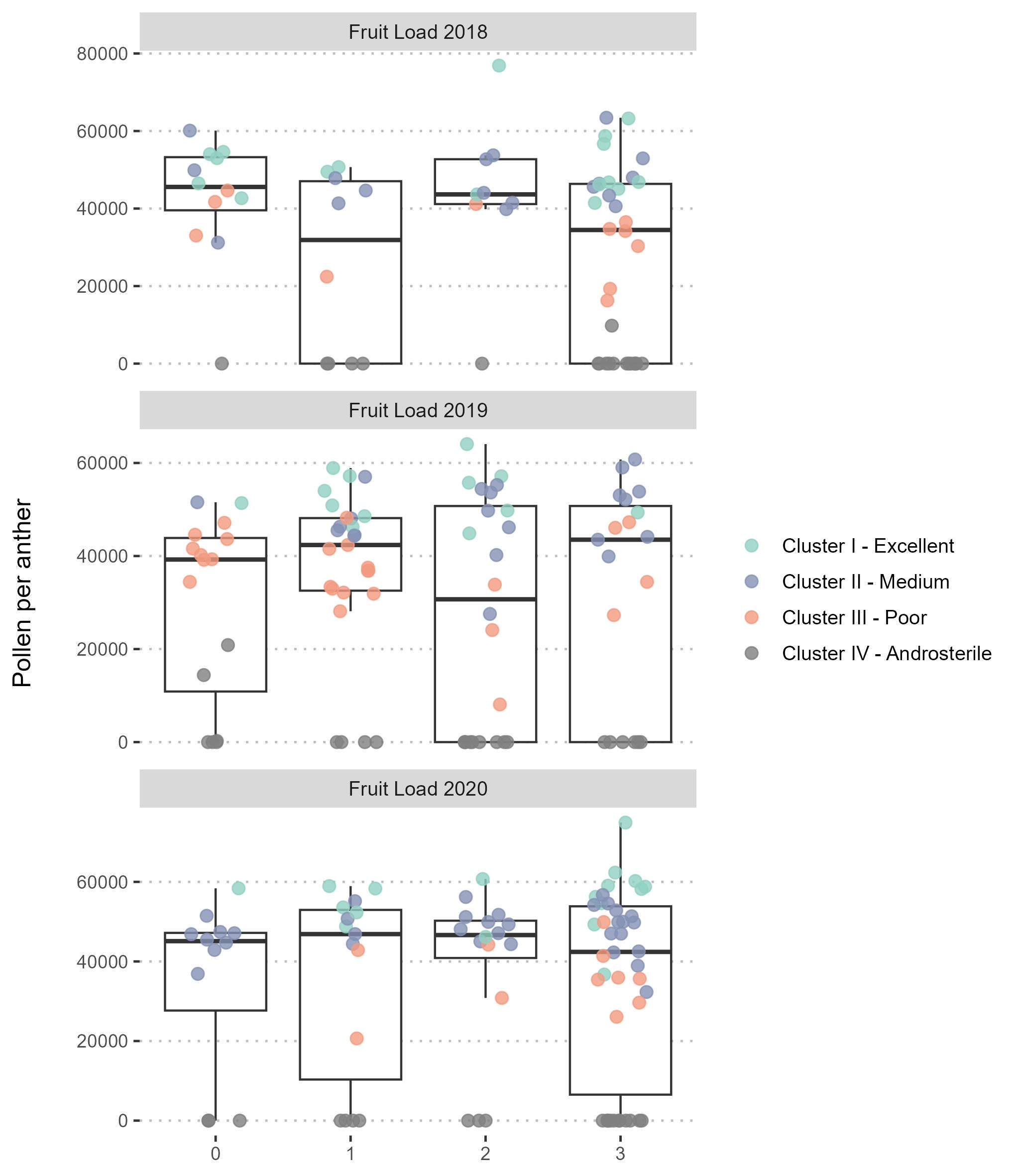

Supplement: mcad163_suppl_Supplementary_Figure_S1 [file mcad163_suppl_supplementary_figure_s1.jpeg]
