# Supplementary material for: Pollen production in olive cultivars and its interannual variability
Source: Ann Bot. 2023 Oct 19;132(6):1145–58. doi: 10.1093/aob/mcad163 (PMC10809056; doi:10.1093/aob/mcad163)
Supplement: mcad163_suppl_Supplementary_Table_S1 [file mcad163_suppl_supplementary_table_s1.docx]

Table S1. Pollen production and summary of the air temperatures and precipitation during the months prior to flowering in the three evaluated years.

| **Year** | **Month** | **Tmin (ºC)** | **Tmax (ºC)** | **Tmean (Cº)** | **Prec (mm)** | **Pollen grains production** |
| --- | --- | --- | --- | --- | --- | --- |
| 2019 | Jan | 1.3 | 14.9 | 8.0 | 33 |  |
|  | Feb | 3.0 | 19.0 | 11.1 | 5 |  |
|  | Mar | 5.7 | 22.3 | 14.0 | 17 |  |
|  | Apr | 8.0 | 21.2 | 14.6 | 56 |  |
|  | Cummulated |  |  |  | 111 |  |
| Average |  | 6.2 | 21.5 | 13.9 | 22 | 38,561 |
|  |  |  |  |  |  |  |
| 2020 | Jan | 4.2 | 14.4 | 9.3 | 41 |  |
|  | Feb | 6.4 | 20.5 | 13.4 | 1 |  |
|  | Mar | 7.3 | 20.3 | 13.8 | 46 |  |
|  | Apr | 10.6 | 21.2 | 16.0 | 75 |  |
|  | Cummulated |  |  |  | 163 |  |
| Average |  | 8.4 | 21.2 | 14.8 | 37 | 34,768 |
|  |  |  |  |  |  |  |
| 2021 | Jan | 2.7 | 13.1 | 7.8 | 63 |  |
|  | Feb | 7.0 | 17.2 | 12.1 | 43 |  |
|  | Mar | 5.8 | 20.8 | 13.4 | 16 |  |
|  | Apr | 10.4 | 22.8 | 16.6 | 60 |  |
|  | Cummulated |  |  |  | 182 |  |
| Average |  | 7.4 | 20.5 | 14.0 | 38 | 42,036 |

Tmin: Average minimum temperature

Tmax: Average maximum temperature

Tmean: Average mean temperature

Prec: Accumulated rainfall
